# Human desmoid fibroblasts: matrix metalloproteinases, their inhibitors and modulation by Toremifene

**DOI:** 10.1186/1471-2407-5-22

**Published:** 2005-03-01

**Authors:** Chiara Balducci, Cinzia Lilli, Giordano Stabellini, Lorella Marinucci, Giammario Giustozzi, Alessio Becchetti, Lucio Cagini, Paola Locci

**Affiliations:** 1 Department of Experimental Medicine and Biochemical Science, University of Perugia, Perugia, Italy; 2 Department of Human Anatomy, University of Milan, Milan, Italy; 3 Department of Surgery, University of Perugia, Perugia, Italy

## Abstract

**Background:**

Desmoid tumour is a benign, non metastasising neoplasm characterised by an elevated deposition of organic macromolecules in the extracellular matrix (ECM). The matrix metalloproteinases (MMPs) are a family of zinc-dependent proteinases involved in the degradation of ECM macromolecules. The MMPs and their natural inhibitors (TIMPs) have been implicated in tumour growth, invasion and metastasis. In this study we provide evidence that the in vitro cultured cell line from desmoid tumour accumulates more collagen fibres in the ECM than healthy fibroblasts.

**Methods:**

We investigated collagen accumulation by ^3^H-thymidine incorporation, MMP expression by substrate gel zymography and TIMP expression by Western blot analysis.

**Results:**

Desmoid fibroblasts showed a reduction in MMP activity and an increase of type I and III collagen and TIMPs compared to normal fibroblasts.

**Conclusion:**

The increase in collagen in desmoid fibroblasts was due to inhibited collagen degradation (reduction of MMP activity) rather than to increased collagen synthesis. Adding toremifene, an anti-estrogen triphenylethylene derivate, to desmoid fibroblasts reduced collagen accumulation by decreasing mRNA expression and increasing collagen degradation.

## Background

Desmoid tumours, which are frequently observed in Gardner's syndrome, are rare, slow-growing, histologically benign tumours caused by autosomal dominant gene mutation [1,2]. They are, however, locally aggressive, compress surrounding structures and show frequent recurrences after surgical removal. Desmoid cells are characterized by abundant deposition of organic macromolecules in the extracellular matrix (ECM), by enhanced transforming growth factor β_1 _(TGFβ_1_) gene expression and increased protein secretion [3]. Cell proliferation, angiogenesis and the accumulation of ECM macromolecules are all facilitated by tumour cell production of TGFβ_1 _[3-6]. All components of ECM are degraded by matrix metalloproteinases (MMPs), a family of zinc-dependent neutral endopeptidases [7]. Two types of MMPs are required for dissolution of interstitial collagen: collagenases and gelatinases [8]. Collagenase-1 (MMP-1), collagenase-2 (MMP-8) and collagenase-3 (MMP-13) are the principal secreted neutral proteinases that initiate degradation of native fibrillar collagens of type I, II, III and V. They all cleave fibrillar collagens at a specific site, resulting in the generation of N-terminal 3/4 and C-terminal 1/4 fragments, which are further degraded by gelatinases [7,9,10]. Gelatinase-A (MMP-2) is expressed by several types of cells, especially fibroblasts, whereas gelatinase-B (MMP-9) is restricted to epithelial cells. MMP-2 and MMP-9 are thought to play major roles in the final degradation of fibrillar collagens after first cleavage by collagenases and denaturation [11]. MMP-2 also cleaves native type I collagen to N-terminal 3/4 and C-terminal 1/4 fragments which are identical to those generated by collagenases [12]. Several different tissue inhibitors of matrix metalloproteinases (TIMPs; TIMP-1 to TIMP-4) have been identified as the major natural inhibitors of MMPs [13]. TIMP-1 and TIMP-2 inhibit the activity of most MMPs [11]. Expression of TIMP-1 is up-regulated at the transcription level by various growth factors such as TGFβ_1_, whereas TIMP-2 is largely expressed constitutively by cultured cells [14]. Our previous studies showed desmoid fibroblasts enhanced deposition of organic macromolecules in the ECM and TGFβ_1 _secretion [3]. Even if desmoid cells do not have estrogen receptors [3], adding toremifene, an antiestrogenic triphenylethylene derivate, decreased TGFβ_1 _production and ECM macromolecule accumulation through a mechanism of action that still remains unclear [3,6,15,16]. The present study investigates the rule of MMPs and TIMPs in the desmoid tumour and describes, for the first time, the effects of toremifene on MMPs and TIMPs. The results provided evidence that toremifene reduced ECM accumulation by decreasing collagen synthesis and increasing collagen degradation.

## Methods

### Antiestrogen

Toremifene (4-chloro-1,2-diphenyl-1-{4-[2-(N,N-dimethylamino) ethoxy] phenyl}-1-butene) citrate was purchased from Farmos (Farmos Group Ltd, Finland).

### Cell cultures

Fibroblast cell lines were obtained from patients with Gardner's syndrome and were provided by NIGMS (Camden, N.J.). The GMO 6965 cell line was obtained from phenotypically healthy fibroblasts, and the GMO 6888 cell line was obtained from desmoid fibroblasts. All cell lines were cultured in Eagle's minimal essential medium (MEM) (Sigma, St. Louis, MO) supplemented with 20% fetal bovine serum (FBS) (GIBCO-Invitrogen, Basel, Switzerland), 2% non-essential amino acids (GIBCO), 2 mM L-glutamine, 100 U/ml penicillin and 100 U/ml streptomycin in a humidified 5% CO_2 _atmosphere at 37°C. Confluent cultures were obtained after 48 h of in vitro maintenance. The cells were cultured for 12 h in MEM. The medium was then discarded to avoid serum factor contamination. Toremifene was dissolved in ethanol and all the cultures were maintained in MEM containing ethanol or MEM containing toremifne in ethanol and treated as described below.

### Cell viability

Normal (control) and desmoid fibroblasts were cultured for 24 h in MEM and ethanol or MEM containing 1 μM toremifene in ethanol (final concentration 0.1% v/v). Then 50 μl of sterile 0.4% trypan blue solution (final concentration 0.05%) was added to each culture well; cultures were incubated at 37°C for 15 min. Viable cells (trypan blue negative) and dead cells (trypan blue positive) were counted by a Burker chamber.

### Collagen synthesis

Confluent cultures of normal (GMO 6965) and desmoid fibroblasts (GMO 6888) were cultured for 3, 24 and 48 h in MEM without serum supplemented L-ascorbic acid (50 μg/ml), β-aminopropionitrile fumarate (50 μg/ml), 8 μCi/ml of ^3^H-proline (specific activity 35 Ci/mmole, Amersham, Freiburg, Germany) in the presence or absence of 1 μM toremifene. In a second set of experiments desmoid fibroblasts were cultured in MEM supplemented with L-ascorbic acid (50 μg/ml), β-aminopropionitrile fumarate (50 μg/ml) for 48 h with or without toremifene. ^3^H-proline was added for 48 h, for the last 24 and for the last 3 h. At the end of the labelling period collagen was extracted using the method of Webster and Harvey [17]. Samples were digested with pepsin (1 mg/ml) in mild agitation overnight at 4°C. Collagen was precipitated and redissolved in 500 μl cold acetic acid 0.5 M. Total radioactivity was counted in a liquid scintillation counter and expressed as cpm/μg protein.

### Northern blot analysis of procollagen α_1 _(I)

Total RNA was isolated from confluent cultures of normal and desmoid fibroblasts maintained for 48 h in MEM alone or supplemented with 1 μM toremifene using the method of Chomczynski and Sacchi [18]. For Northern blot analysis equal amounts of total RNA (20 μg) were electrophoresed on 1% agarose gel containing 0,66 M formaldehyde and transferred on to nylon filters (Hybond N, Amersham). Before blotting, the gel was rinsed in water for 15 min at room temperature and then in 20X SSC (1 X SSC is 0.15 M sodium chloride, 0.015 M sodium citrate, pH 7) for 10 min. Blots were pre-hybridised in 20 ml of a cocktail containing 1 mM EDTA pH 8, 0.25 M Na_2_HPO_4 _pH 7.2 and 7% sodium dodecyl sulfate (SDS) for 4 h at 65°C. Probes were labelled with [α-^32^P] dCTP (3000 Ci/mM) by random priming (Amersham RPN 1601). Hybridisation was performed at 65°C overnight using 10^6^cpm/ml probe in the same buffer used for pre-hybridisation. After hybridisation, the nylon membrane was washed twice in 1 mM EDTA pH 8, 20 mM Na_2_HPO_4 _pH 7.2 and 5% SDS at 65°C (5 min each) and then washed twice with 1 mM EDTA pH 8, 20 mM Na_2_HPO_4 _pH 7.2 and 1% SDS at 65°C (5 min each). The filters were stripped and re-hybridised with a GAPDH probe to assess blot loading. For autoradiography the membranes were exposed to Kodak X-Omat film (Rochester, NY) at -80°C for 1 day. Autoradiographies were analysed by computerised scanning densitometry. Results are expressed as the ratio of procollagen α1(I)/control GAPDH densitometry signals.

### cDNA probes

A 670 bp Eco RI-Hind III cDNA fragment from human pro-collagen α1(I) and a 1.3 kb PstI cDNA fragment from rat glyceraldehyde-3-phosphate dehydrogenase were used as probes in hybridisation [18].

### Collagenase activity

Collagenase activity was determined using the method of Khorramizadeh et al., [19]. Confluent normal and desmoid fibroblasts were washed with MEM and cultured for 48 h in MEM or in MEM containing 1 μM toremifene. Proteins in the medium were precipitated by ammonium sulphate 65% w/v; precipitates were collected by centrifugation, dissolved in assay buffer (0.05 M Tris-HCl, 0.2 M NaCl, 5 mM CaCl_2_, 0.02% sodium azide, pH 7.4) and then dialysed overnight against the same buffer. The latent procollagenase was activated with trypsin (10 μg/ml) and then the trypsin was inactivated with soybean trypsin inhibitor (100 μg/ml). Acetic acid soluble type I collagen (25 μl of a solution 2 mg/ml) from bovine skin was incubated with the activated collagenase solution for 24 h. The products of the collagen digestion were separated by electrophoresis using 6% acrylamide gel containing SDS. The gels were stained with 0.25% Coomassie brilliant blue G-250 (50% methanol, 10% acetic acid), destained appropriately (40% methanol, 10% acetic acid) and fixed (5% methanol, 7.5% acetic acid). The digestion products were quantified with a computerised scanner.

### Preparation of conditioned media (CM)

Confluent normal (GMO 6965) and desmoid (GMO 6888) fibroblasts were washed with 0.9% NaCl and cultured for 12 h in serum-free MEM. This medium was discarded to avoid contamination by serum factors and cells were cultured for the next 24 h in MEM and ethanol (control) or in MEM containing 1 μM toremifene in ethanol (final ethanol concentration 0.1% v/v). Conditioned media (CM) were collected and centrifuged for 10 min at 350 *g *to remove cell debris, dialysed against bidistilled water for 24 h, lyophilised and used for zymography and Western blot analysis as described below.

### Collagen and gelatin zymography

CM were analysed for gelatinases and collagenases by zymography. Samples were separated under non reducing condition on 6% polyacrylamide gels containing 1 mg/ml of gelatin (Sigma Chemical, St Louis; MO, USA) or 1 mg/ml collagen (Sigma) [20]. In one set of samples the proenzymatic forms were activated using 2 mM aminophenylmercuric acetate (APMA) for 1 h at 37°C. Samples were lyophilised and resuspended in Tris-HCl 0.4 M pH 6.8, SDS 5%, 20% glycerol and 0.03% bromophenol blue. Gels were loaded with 8 μg protein per sample or with 2 μg trypsin and run under Laemmli conditions [21]. After electrophoresis, gels were washed twice in 200 ml of 2.5% Triton X-100 (30 min each) under constant mechanical stirring and incubated in 50 mM Tris-HCl pH 7.5, 5 mM CaCl_2_, 0.02% Brij-35 and 200 mM NaCl at 37°C for 24 h. Gels were stained with Coomassie brilliant blue G-250. Proteinase activity, observed as cleared (unstained) regions, was converted to dark regions to better observation of bands.

### Western-blot analysis

CM were analysed for type I and type III collagen, MMP-1, MMP-2, MMP-9, TIMP-1 and TIMP-2 by Western blotting using specific monoclonal antibodies. Aliquots of CM, containing 50 μg of proteins, were separated on SDS-10% polyacrylamide gels under reducing conditions and transferred on to a nitrocellulose membrane. The membrane was blocked with blocking solution (5% w/v dried skimmed-milk powder in TBS 1X, 2 h at room temperature) and incubated with the specific monoclonal antibody in antibody solution (1% w/v dried skimmed-milk powder in TBS 1X, 2 h at room temperature). Bound antibody was detected with a sheep anti-mouse peroxidase-conjugated antibody in antibody solution. Western analysis was performed using chemiluminescence reagents from Amersham Pharmacia Biotech.

### Protein determination

Protein concentrations were determined by the Lowry assay [22] of aliquots of cell lysate.

### Statistical analysis

In some experiments, statistical analysis was performed using Student's *t*-test. Data are expressed as the means ± SD of four determinations. In other experiments, the results are reported as means ± SD of three separate experiments, each performed in quadruplicate. Statistical analysis was performed by Student's two-tailed *t*-test and by analysis of variance (ANOVA) followed by Sheffe F-test.

## Results

### Cell viability

The amount of dead cells and viable cells in normal fibroblasts, desmoid fibroblasts and desmoid fibroblasts plus toremifene was evaluated after 24 h of in vitro maintenance in the presence of trypan blue (Table 1). Granted that the number of intact viable cells was high in all the experimental conditions, desmoid fibroblasts had the highest number of cells/culture and the lowest percentage of dead cells (0.0014%). Treatment of desmoid fibroblasts with toremifene enhanced the percentage of dead cells (0.011%) which, nevertheless, remained lower than in normal fibroblasts (0.025%.).

### Effects of toremifene on collagen synthesis

Collagen synthesis was evaluated after 3, 24 and 48 h of in vitro maintenance in the presence of ^3^H-proline (Table 2). No significant difference was observed after 3 hours culture. After 24 and 48 h culture collagen production was significantly higher in desmoid than in normal fibroblasts, in both the cellular and extracellular compartments. The increase was 1.4 fold in the cells and 1.8 fold in the medium after 24 h; 1.3 fold in the cells and 1.8 fold in the medium after 48 h. Adding toremifene significantly decreased collagen synthesis at 24 and at 48 h. The reduction was greater after 48 h (42% in the cells and 38% in the medium). In a second set of experiments desmoid fibroblasts were cultured for 48 h with or without toremifene. The radiolabelled precursor was added for 48 h, in the last 24 h and in the last 3 h (Table 3). Treatment with toremifene had an inhibitory effect at all times. The decrease in total collagen (cells + media) in desmoid fibroblasts treated with toremifene was 28% in the presence of ^3^H-proline for 48 h, 46% and 52% respectively in the presence of ^3^H-proline in the last 24 or 3 h of in vitro maintenance (Table 3).

### Procollagen α_1 _(I) mRNA expression

**Figure 1:**
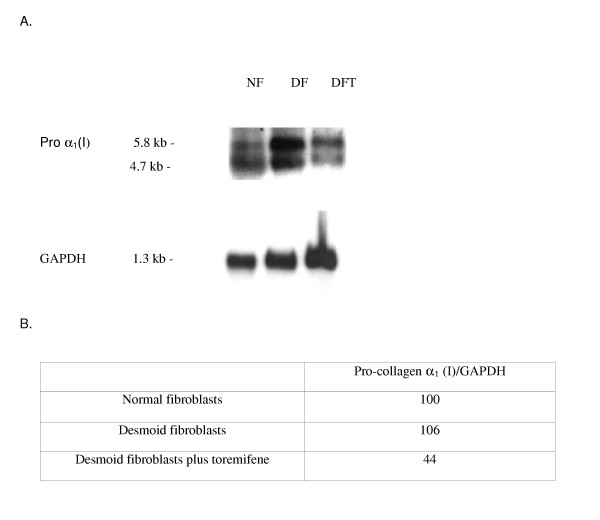
**Expression of procollagen α_1 _(I) and glyceraldehydes-3-phosphate dehydrogenase (GAPDH) mRNA in normal and desmoid fibroblasts. **Panel A: NF: normal fibroblasts; DF: desmoid fibroblasts; DFT: desmoid fibroblasts plus toremifene. Similar results were obtained in four separate experiments. In panel B the absolute counts, obtained by densitometric analysis, were converted to percentages of control value, assuming the untreated level of normal fibroblasts as 100%.

Northern blots were performed to analyse procollagen α_1 _(I) mRNA level in normal and desmoid fibroblasts (Fig. 1). Relative densitometric units were normalised to GAPDH mRNA levels. Normal and desmoid fibroblasts exhibited no significant differences in the steady-state mRNA levels for procollagen α_1 _(I). Toremifene down regulated procollagen mRNA expression by 58% in desmoid cells.

### Western-blot analysis of type I and III collagen

**Figure 2:**
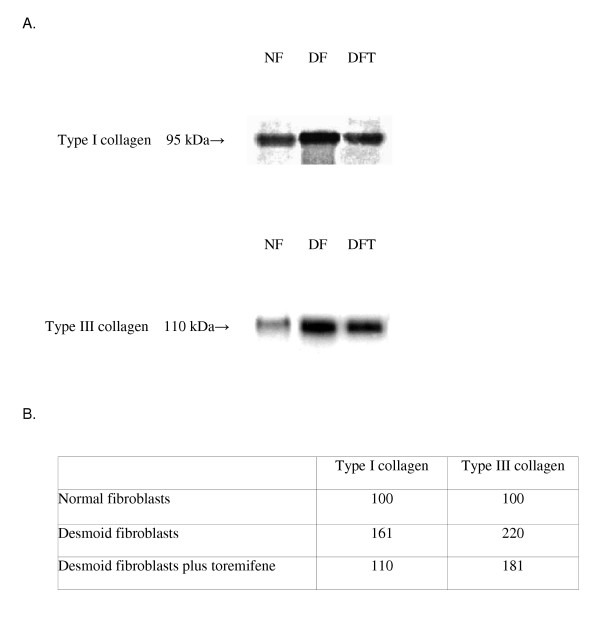
**Western blot analysis of type I and III collagen secreted into the medium by normal and desmoid fibroblasts. **Panel A. The samples are as follow: NF normal fibroblasts; DF desmoid fibroblasts; DFT desmoid fibroblasts plus toremifene. Similar results were obtained in four separate experiments. Panel B. The absolute counts, obtained by densitometric analysis, were converted to percentages assuming the level of normal fibroblasts as 100%.

Media from normal and desmoid fibroblasts with or without toremifene were analysed by Western blotting to evaluate the presence of type I and III collagen using specific monoclonal antibodies (Fig. 2). Densitometric tracing of the autoradiograms quantified collagen secretion. Desmoid fibroblasts secreted much more type I (1.6 fold) and III (2.2 fold) collagen than normal cells. Toremifene reduced type I and III collagen by 31% and 18% respectively in desmoid fibroblasts.

### Collagenase activity

**Figure 3:**
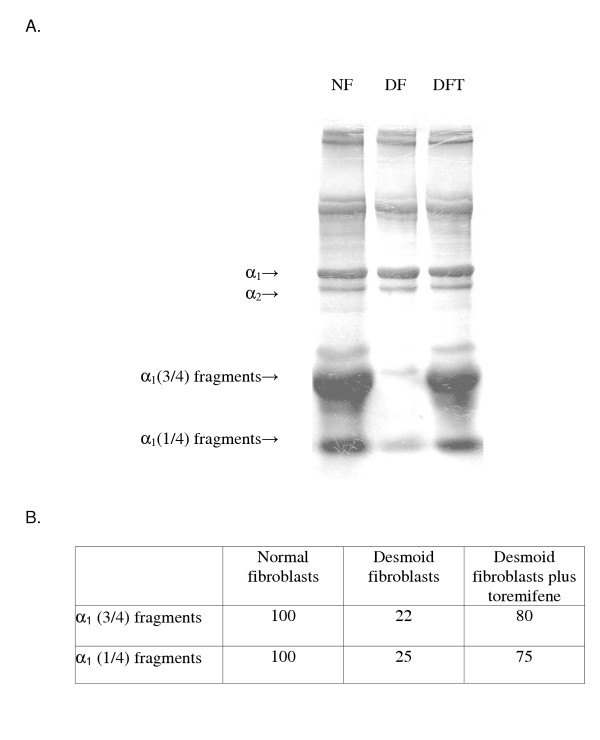
**Collagenase activity in media derived from normal, desmoid fibroblasts and desmoid fibroblasts plus toremifene. **Panel A. NF: normal fibroblasts; DF: desmoid fibroblasts; DFT: desmoid fibroblasts plus toremifene. Similar results were obtained in four separate experiments. In panel B the quantity of 3/4 and 1/4 fragments of digested collagen was determined by densitometric analysis. The absolute counts were converted to percentages assuming the level of normal fibroblasts as 100%.

Collagenases, from ammonium sulphate-precipitated proteins of media of normal fibroblasts, desmoid fibroblasts and desmoid fibroblasts treated with toremifene, were incubated with soluble collagen and the digested products were evaluated by gel electrophoresis. Collagenases in the medium of normal fibroblasts digested more α_1 _and α_2 _chains of type I collagen into their corresponding 3/4 and 1/4 fragments than the collagenase in desmoid fibroblasts (Fig. 3). When band staining intensity was quantified by densitometry, the abundance of the 3/4 and 1/4 products of collagenase digestion was significantly greater in normal than in desmoid fibroblasts. Adding toremifene to desmoid fibroblasts markedly increased collagenase activity as shown by the increased amount of 3/4 and 1/4 fragments of α_1 _and α_2 _chains (Fig. 3).

### Collagen and gelatin zymography

**Figure 4:**
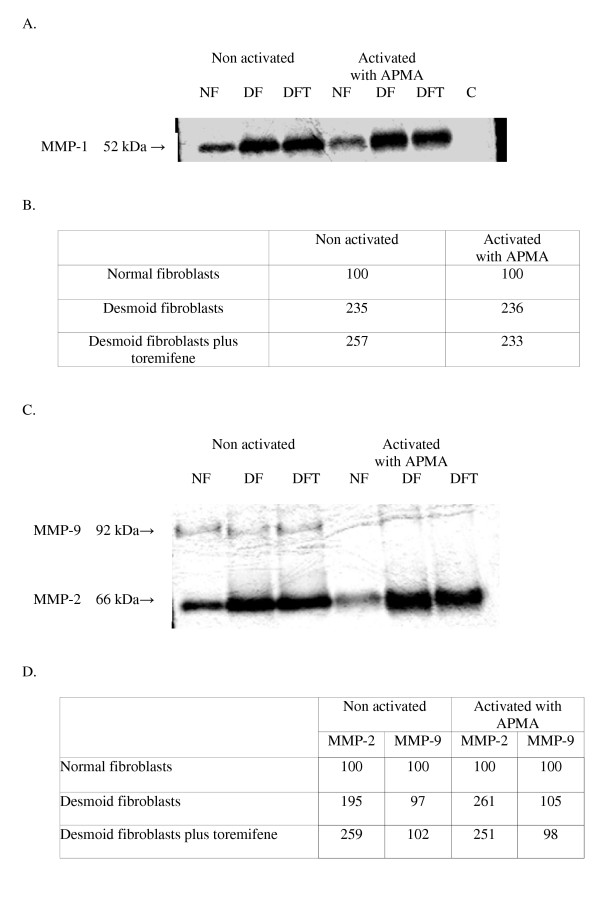
**Zymogram of media from normal fibroblasts, desmoid fibroblasts, desmoid fibroblasts plus toremifene. **Collagen zymogram. One set of samples was treated with APMA to activate the proenzymatic forms. Panel A. NF: normal fibroblasts, DF: desmoid fibroblasts, DFT: desmoid fibroblasts plus toremifene. In the same zymogram an aliquot of samples was activated with APMA: NF: normal fibroblasts, DF: desmoid fibroblasts, DFT: desmoid fibroblasts plus toremifene, C: trypsin. Similar results were obtained in four separate experiments. Panel B. The absolute counts, obtained by densitometric analysis, were converted to percentages assuming the level of normal fibroblasts as 100%. Gelatin zymogram. One set of samples was treated with APMA to activate the proenzymatic forms. Panel C. NF: normal fibroblasts, DF: desmoid fibroblasts, DFT: desmoid fibroblasts plus toremifene. In the same zymogram an aliquot of samples was activated with APMA: NF: normal fibroblasts, DF: desmoid fibroblasts, DFT: desmoid fibroblasts plus toremifene. Similar results were obtained in four separate experiments. Panel D. The absolute counts, obtained by densitometric analysis, were converted to percentages assuming the level of normal fibroblasts as 100%.

Collagen and gelatin zymograms dosed the enzymatic activity of collagenases and gelatinases. Collagen zymogram, reported in Fig. 4 (panel A and B), showed the samples produced a band of 52 kDa corresponding to MMP-1. Densitometric analysis of the counts, assuming the value of normal fibroblasts as 100%, demonstrated 2.3 fold increase in the 52 kDa collagenase activity in desmoid fibroblasts. When desmoid fibroblasts were treated with toremifene, the level of collagenase activity in the media was only minimally affected. No bands were present in trypsin (Fig. 4, panel A, line C), which can degrade gelatin but not collagen, confirming that collagen has been degraded in panel A. The gelatin zymogram (Fig. 4, panel C and D) showed two bands, one of 92 kDa corresponding to MMP-9, the other of 66 kDa corresponding to MMP-2. Desmoid fibroblasts produced the same amount of MMP-9, and larger (about 2 fold) amounts of MMP-2, than normal fibroblasts Adding toremifene to desmoid fibroblasts increased only MMP-2 activity by about 1.32 fold. To verify whether the bands detected in the collagen and gelatin zymography were due only to MMPs, two control gels were washed and incubated in buffers containing 10 mM EDTA. No bands were detected after this treatment, which indicated that the bands obtained in collagen and gelatin zymographies were entirely due to MMP activity. Toremifene addition to desmoid cells was accompanied by no changes in gelatinase activity. One set of samples in collagen and gelatin zymograms was treated with APMA to activate the proenzymes. Activation of the proenzymatic form had no significant effects on collagenase activity (Fig. 4, panel B), but enhanced gelatinase activity in desmoid fibroblasts (Fig. 4, panel D).

### Western-blot analysis of MMP-1, MMP-2, MMP-9

**Figure 5:**
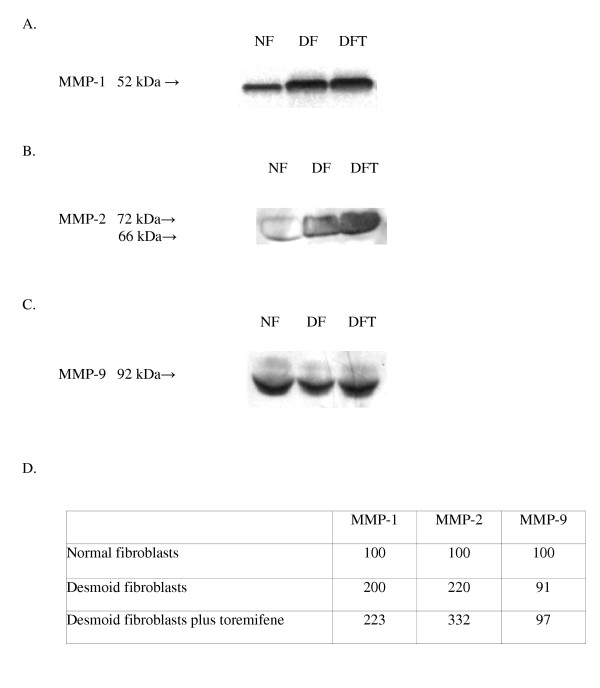
**Western blot analysis of MMP-1, MMP-2, MMP-9 secreted into the medium. **Panel A: MMP-1; panel B: MMP-2; panel C: MMP-9. The samples are as follows: NF, normal fibroblasts; DF, desmoid fibroblasts; DFT, desmoid fibroblasts plus toremifene. Similar results were obtained in four separate experiments. Panel D. The absolute counts, obtained by densitometric analysis, were converted to percentages assuming the level of normal fibroblasts as 100%. ND = not determined.

The presence of MMP-1, MMP-2, MMP-9 in the media of normal fibroblasts, desmoid fibroblasts and desmoid fibroblasts plus toremifene was evaluated by Western-blot analysis using specific monoclonal antibodies (Fig. 5). Western blot analysis of MMP-1 (Fig. 5, panel A) showed that the amount of the protein was higher in desmoid (2 fold) and in desmoid than in normal fibroblasts (2 fold). Toremifene exhibited no significant increase of MMP-1 in desmoid cells (about 2.2 fold). MMP-2 (Fig. 5, panel B) showed two bands, the first due to the proenzymatic form (72 kDa) and the second to the active form (66 kDa). MMP-2 was significantly increased in desmoid fibroblasts (2.2 fold) and even more in desmoid fibroblasts plus toremifene (3.2 fold) compared with normal fibroblasts. No significant differences emerged in the production of MMP-9 (Fig. 5, panel C).

### Western-blot analysis of TIMP-1 and TIMP-2

**Figure 6:**
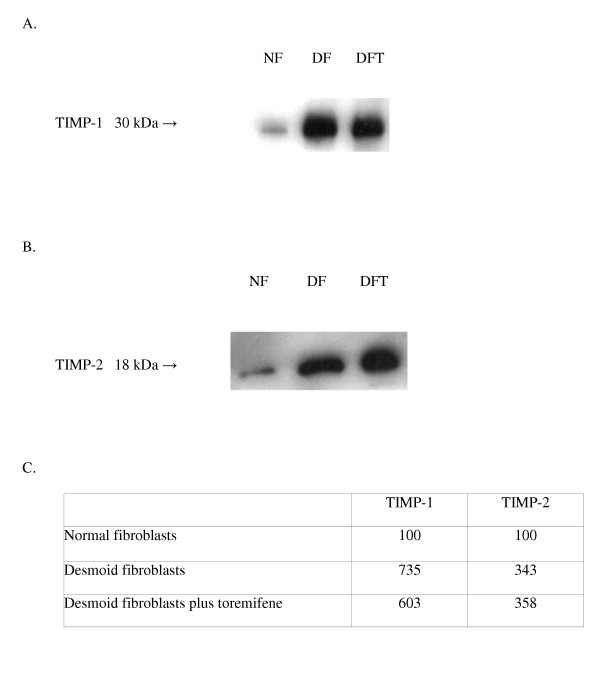
**Western blot analysis of TIMP-1 and TIMP-2 secreted into the medium. **Panel A; TIMP-1. Panel B; TIMP-2. The samples are as follows: NF, normal fibroblasts; DF, desmoid fibroblasts; DFT, desmoid fibroblasts plus toremifene. Similar results were obtained in four separate experiments. Panel C. The absolute counts, obtained by densitometric analysis, were converted to percentages assuming the level of normal fibroblasts as 100%.

Western blot analysis showed that desmoid fibroblasts produced about 7.2 and 3.4 fold TIMP-1 (Fig. 6, panel A) and TIMP-2 respectively (Fig. 6, panel B) than normal fibroblasts. Adding toremifene to desmoid fibroblasts decreased TIMP-1 by 18%, but had no effect on TIMP-2.

## Discussion

Desmoid tumour is a benign non-invasive and non-metastasising neoplasm with an abnormal ECM macromolecule deposition which is stimulated by TGFβ_1 _[3,23,24]. The regulation of extracellular matrix dynamics is clearly complicated, involving a balance between the deposition of structural components such as collagen and their degradation by MMPs, i.e. collagenases and gelatinases. MMP activity is itself regulated by a variety of mechanisms, including a requirement for enzyme modification to elicit maximal enzymatic activity and the activity of specific TIMPs [25]. There is now evidence that desmoid cells undergo dramatic clinical response to toremifene, implying the drug has a direct effect upon fibroblasts. Our previous studies showed that toremifene significantly inhibited TGFβ_1 _activity which was six fold higher in desmoid than in normal fibroblasts [3]. As desmoid tumour is also associated with abnormal collagen production [26], in the present study we examined the rate of collagen synthesis and degradation in the presence or absence of toremifene. In our experimental conditions, type I and III collagen accumulation in the intra- and extra-cellular compartments showed no differences after 3 h of in vitro maintenance, but increased significantly more after 24 and 48 h in desmoid fibroblasts than in normal fibroblasts. No increase in collagen after 3 hours suggests its accumulation in desmoid fibroblasts is due to inhibition of degradation rather than to increased synthesis. The results are confirmed by procollagen α_1 _(I) gene expression, which showed mRNA levels were only lower in desmoid cells treated with toremifene. Normal and desmoid fibroblasts expressed different amounts of MMPs. Several studies suggest that MMPs are over-expressed in malignant tumour progression and facilitate both local tumour invasion and metastasis [27,28]. Different MMPs may play distinct roles at different stages of tumour development [29]. They may form a network, in which a single MMP is crucial for the cleavage of certain native or partially degraded matrix components and for the activation of other latent MMPs. MMP-1 plays a pivotal role in cancer progression and poor prognosis in colon-rectal, oesophageal and gastric cancer has been correlated with high MMP-1 expression [25,30]. Nishiota [31] showed MMP-1 is expressed more strongly in the cancer front of invasion. MMP-2 is increased in cancer tissue and its over-expression is correlated with tumour-related basement membrane degradation and vascular invasion [32,33]. Therefore inhibition of the expression or activity of only one MMP could potentially reduce peritumoural proteolytic activity and tumour invasion [34]. In this study we investigated the metalloproteinases most involved in type I collagen degradation, i.e. MMP-1 (collagenase-1), MMP-2 (gelatinase-A) and MMP-9 (gelatinase-B) and their natural inhibitors TIMP-1 and TIMP-2 [11]. Moreover TIMP-2 is 10-fold more potent than TIMP-1 against MMP-2 [11] which is involved either in the final degradation of native collagen or in the initial degradation cleaving native type I collagen to 3/4 and 1/4 fragments identical to those generated by MMP-1 [12]. Using Western blot we showed no differences in MMP-9 production, while MMP-1 and MMP-2 were higher in desmoid than in normal fibroblasts. Collagen and gelatin zymograms, in which the proteolytic enzymes were separated from TIMPs before the assay, proved the activities of collagenase MMP-1 and gelatinase MMP-2, as dosed in conditioned media, were higher in desmoid than in normal fibroblasts. However, collagenase activity, in the presence of TIMPs, was reduced in desmoid compared to normal fibroblasts as shown by the lower amount of 3/4 and 1/4 fragments of fibrillar collagen in desmoid cells. Together these results indicated the higher MMP-1 and MMP-2 activity in desmoid cells was masked by a 7-fold increase in TIMP-1 and a 3-fold increase in TIMP-2. TIMP-1 is a potent inhibitor of apoptosis in many cells types, its up-regulation protects the cells against apoptotic stimuli [35]; hence, greater number of viable cells in desmoid tumour.

Upregulation in both inhibitors of MMPs may explain why the Desmoid tumour is characterised by an abundant deposition of ECM macromolecules and is neither malignant nor invasive. Toremifene addition to desmoid fibroblasts reduced the accumulation of collagen fibres but its mechanism of action remains unclear. Toremifene increased MMP-1 and MMP-2 activity by 8% and 25% respectively and decreased TIMP-1 by 18%. Despite these modest effects type I collagen degradation in 3/4 and 1/4 fragments increased almost 4-fold.

## Conclusion

Our previous studies showed that TGFβ_1 _was 6-fold higher in desmoid than in normal fibroblasts and that toremifene significantly reduced TGFβ_1 _activity and TGFβ_1 _membrane-receptors [3]. So the effects of toremifene on MMPs and TIMPs could be linked to its effects on TGFβ_1 _because the growth factor enhances organic macromolecule accumulation in the ECM via a reduction in MMP-1 and MMP-2 [36] and an increase in TIMP-1 [37], so favouring tumour mass growth through an inhibition of ECM macromolecule degradation. In the light of these data the reduction of organic macromolecules in the ECM in the presence of toremifene can be ascribed to its inhibition not only of collagen synthesis, but also of TGFβ_1 _activity. Further studies on the regulation of MMP activities may clarify the role of toremifene on ECM degradation and provide important clues about pathogenesis of desmoid tumour.

## Competing interests

The author(s) declare that they have no competing interests.

## Authors' contributions

CB carried out collagen synthesis, collagenase activity and drafted the manuscript.

CL and GB participated in the design of the study and carried out Northern blot analysis.

LM and GG carried out RT-PCR, zimography and oestrogen receptor assay.

AB and LC carried out Western blot analysis and performed the statistical analysis.

PL conceived of the study, and participated in its design and coordination.

All authors read and approved the final manuscript.

## Pre-publication history

The pre-publication history for this paper can be accessed here:



## Figures and Tables

**Table 1:** Cell viability

	Viable cells	Dead cells	% of dead cells
Normal fibroblasts	1,224,805 ± 56,280	308 ± 26	0.025
Desmoid fibroblasts	1,636,710 ± 84,105*	29 ± 4	0.0017
Desmoid fibroblasts + toremifene	1,393,900 ± 86,400	161 ± 15	0.011

Fibroblasts were cultured for 24 h in MEM containing ethanol with or without toremifene. Number of viable and dead cells/culture after exposure to trypan blue for 15 min. The values are the means ± SD of five cultures. *Significance vs normal fibroblasts: P < 0.01.

**Table 2:** ^3^H-proline incorporation into collagen fibres

	Cells	Media
	3 h	

Normal fibroblasts	1,060 ± 85	998 ± 94
Desmoid fibroblasts	885 ± 60	991 ± 39
Desmoid fibroblasts + toremifene	1,112 ± 134	837 ± 36

	24 h	

Normal fibroblasts	2,313 ± 99	6,523 ± 388
Desmoid fibroblasts	3,340 ± 205*	11,641 ± 421*
Desmoid fibroblasts + toremifene	2,286 ± 124°°	9,962 ± 644°°

	48 h	

Normal fibroblasts	4,262 ± 280	17,955 ± 1,073
Desmoid fibroblasts	5,649 ± 103*	32,988 ± 1,321*
Desmoid fibroblasts + toremifene	3,295 ± 261°	20,407 ± 980°

Fibroblasts were cultured for 3, 24 and 48 h in MEM containing ^3^H-proline with or without toremifene. Total radioactivity value is expressed in cpm/mg protein and represents the mean ± SD of three determinations, each in quadruplicate. The statistical analysis was performed with analysis of variance (ANOVA). Differences vs normal fibroblasts: F-test significant at *99%; differences vs desmoid fibroblasts: F-test significant at °99%, °°95%.

**Table 3:** ^3^H-proline incorporation into collagen fibres

	Cells	Media	Cells + Media
	48 h		

Desmoid fibroblasts	6,852 ± 290	33,915 ± 2,307	40,767
Desmoid fibroblasts + toremifene	4,243 ± 361*	25,097 ± 814*	29,340
	24 h		
Desmoid fibroblasts	4,252 ± 416	15,423 ± 1,100	19,675
Desmoid fibroblasts + toremifene	1,432 ± 101*	9,221 ± 381*	10,653
	3 h		
Desmoid fibroblasts	1,176 ± 48	1,498 ± 56	2,674
Desmoid fibroblasts + toremifene	534 ± 31*	745 ± 74*	1,279

Fibroblasts were cultured for 48 h in MEM with or without toremifene. ^3^H-proline was added for 48 h, for the last 24 h and for the last 3 h. Total radioactivity value is expressed in cpm/mg protein and represents the mean ± SD of three determinations, each in quadruplicate. The statistical analysis was performed with analysis of variance (ANOVA). Differences vs desmoid fibroblasts: F-test significant at *99%.
